# Effect of web-implemented exercise interventions on depression and anxiety in patients with neurological disorders: a systematic review and meta-analysis

**DOI:** 10.3389/fneur.2023.1225356

**Published:** 2023-07-18

**Authors:** Hanyue Zhang, Rong Wang, Zhenxing Kong, Jingjing Yu, Xiao Hou, Shouwei Zhang

**Affiliations:** ^1^School of Physical Education, Northeast Normal University, Changchun, China; ^2^Key Laboratory of Sports and Physical Health Ministry of Education, Beijing Sport University, Beijing, China; ^3^School of Sport Science, Beijing Sport University, Beijing, China

**Keywords:** web-implemented exercise, neurological disorder, depression, anxiety, mental health

## Abstract

**Introduction:**

Web-implemented exercise intervention is the latest and innovative method to improve people's mental health. Currently, many studies have proven that web-implemented interventions are effective to improve depression and anxiety in adults. However, the influence of different web-implemented exercise interventions on depression and anxiety in patients with neurological disorders is still unclear.

**Objective:**

The study aims to systematically summarize the type and content of web-implemented exercise interventions and quantify the effect of different web-implemented exercise interventions on depression and anxiety in patients with neurological disorders.

**Methods:**

Four literature databases (PubMed, Web of Science, China National Knowledge Infrastructure, and WanFang data) were searched. The literature search considered studies published in English or Chinese before October 13, 2022. Randomized controlled trials (RCTs) that participants accepted web-implemented interventions were included. Two authors independently extracted data and assessed the risk of bias for included studies. Standardized mean differences (SMD) with 95% CI were used to integrate the effect size.

**Results:**

16 RCTs (a total of 963 participants) were included. The results showed that web-implemented exercise intervention had a significant effect on depression (SMD = −0.80; 95% CI, −1.09 to −0.52; *I*^2^ = 75%; *P* < 0.00001) and anxiety (SMD = −0.80; 95% CI, −1.23 to −0.36; *I*^2^ = 75%; *P* = 0.0003) in patients with a neurological disorder. The subgroup analysis showed that the effectiveness of the web-implemented exercise intervention was influenced by several factors, such as web-implemented exercise intervention type, component, and intervention duration.

**Conclusion:**

Web-implemented exercise intervention has a relieving effect on depression and anxiety symptoms in patients with neurological disorders. Additionally, the intervention type, intervention duration, and component can influence the effect size.

**Systematic review registration:**

https://www.crd.york.ac.uk/PROSPERO/#recordDetails, identifier: CRD42023409538.

## Introduction

The Global Burden of Disease (GBD) shows that the impact of neurological disease on global health is grossly underestimated and the prevalence of neurological and is also on the rise (1). Over the past 30 years, neurological disorders have become the leading cause of disability and the second leading cause of death in the world, with a 39% increase in the number of deaths and a 15% increase in the disability-adjusted life years (2). Many studies have shown that these neurological disorders have an evident negative impact on the physical function and quality of life (QOL) of patients with the onset of the disease. For example, multiple sclerosis (MS), as a non-traumatic disabling neurological disorder, can result in a variety of adverse symptoms (e.g., mobility problems, impaired balance, and loss of sensation), which pose an obstacle to people's normal life (e.g., the ability to work and social activities) and ultimately reduce the QOL of patients (3, 4). Other neurological diseases, such as stroke and Parkinson's disease (PD) which have different etiologies, can also lead to alternations in movement and sensation that impair balance and finally reduce the QOL of individuals (5–7). This decrease in QOL may indirectly lead patients to mood swings (8, 9). Except that, the symptoms such as disability and pain caused by neurological disorders can also directly induce several mental-related emotional disorders (e.g., depression and anxiety) (10).

Mental disorders are likely to be the leading cause of disease burden worldwide by 2030 (11). To some extent, compared with the healthy population, psychological disorders like depression and anxiety are more likely to occur in the patients, especially patients who suffer from dysfunction in the neurological system. One study has compared patients with Alzheimer, a degenerative disease of the central nervous system, to healthy people and found that the incidence rate of depression in Alzheimer's patients (67%) is twice as much as that in ordinary people (31%) (12). Another study has also indicated that people with MS have a risk of depression high to 50%, compared to 10–15% in general people (13). In addition, for patients with serious physical illnesses like neurological disorders, mental problems (e.g., depression and anxiety) can also decrease compliance with the treatment plan by increasing the burden of symptoms, thus affecting the treatment effect (14, 15). Hence, an effective intervention that can alleviate the depression and anxiety of patients with neurological disorders is expected.

As a non-medicine and non-invasive intervention, exercise has been proven effective in relieving depression and anxiety (16–18). Compared to pharmacology, exercise interventions are low-cost and can treat depression and anxiety without side effects. For example, one study has compared the effects of a 20-week resistance training exercise intervention and a 20-week pharmacological treatment on the depressive symptoms in PD patients and found that resistance training had a significant effect on reducing depression and improving the QOL, and functionality of PD (19). Besides, Gracizli et al. (20) have conducted a 12-week exercise prescription of a combination of aerobic and resistance training on MS patients and ordinary individuals and found that the combined exercise has a significant benefit in improving balance, QOL, and relieving depressive symptoms. Hence, exercise therapy can be suggested as an effective method to improve both the physical and mental health of patients with nervous system diseases.

In recent years, with the development of electronic technology and the lockdowns caused by COVID-19, more and more people prefer “online exercise.” The web-implemented exercise interventions which support people who do not seek help from health services because of social stigma or transport problems could prescribe a structured exercise program to be carried out at home with varying degrees of supervision, differing from interventions that focus solely on motivating people to exercise (21, 22). Using web-implemented (e.g., online and digital) communication and technology to implement exercise interventions has become a new fashion (23). In China, more than 780 million people exercise through a variety of online platforms. In the COVID-19 pandemic, online exercise interventions have become a focus for doctors and researchers to treat various patients with different symptoms. Because web-implemented tele-exercise intervention can overcome the inconveniences induced by space and time. Doctors can easily use electronic devices (e.g., mobile phones and computers) as a medium to provide patients with exercise-related knowledge and training programs automatically or artificially (24, 25).

Web-implemented exercise can also play an essential role in the improvement of mental health in patients. Turner et al. have evaluated the effect of the web-implemented exercise intervention on the mental health of MS patients. They have provided the 6-month personalized exercise guidance using a remote health system to monitor exercise plans and progress for MS patients in the experimental group and a 6-month self-directed education method for MS patients in the control group. Eventually, they found that 53.3% of MS patients in the experimental group experienced a clinical improvement in depression symptoms, whereas only 9.1% of MS patients in the control group experienced a clinical improvement in depression symptoms (26). Obviously, this web-implemented exercise intervention can reduce the cost of time and space and facilitate behavioral change through monitoring, ultimately bringing benefits for patients in improving physical activity and depression. Besides, another digital intervention, Virtual Reality (VR) exergames, has also been proven effective for neurological patients' activities of daily living and mental health. For example, Lee et al. (27) have demonstrated that VR dance exergames can enhance balance and alleviate depression symptoms of PD patients effectively. Although there is some evidence indicating the effectiveness of certain specific web-implemented exercise interventions on depression and anxiety in patients with different neurological disorders, few studies have intergraded the effects of different web-implemented exercise interventions on mental health in patients with different neurological diseases.

Therefore, the purpose of this study was to summarize various types of web-implemented exercise and integrate the effects of different web-implemented exercise interventions on depression and anxiety in patients with different neurological disorders. It has important value for the development of exercise interventions on mental health, especially for the future expansion of VR exergames and intelligent exercise intervention modes.

## Methods

### Search strategy

This search followed the Preferred Reported Items for Systematic Review and Meta-analysis (PRISMA) guidelines. The protocol was registered on the international prospective register of systematic reviews (http://www.crd.york.ac.uk/PROSPERO), registration number: CRD42023409538. We searched two English databases, including PubMed and Web of Science (WOS), and two Chinese databases, including CNKI and WANFANG Data. All articles were published before October 13, 2022. Table 1 shows the specific search strategy.

**Table 1:** Search strategy.

**Step**	**Search strategy**
#1	“Neurological disorder” OR “stroke” OR “Parkinson's disease” OR “multiple sclerosis” OR “cognitive impairment” [Title/Abstract]
#2	“Virtual Reality” OR “mobile health” OR “exercise” OR “exergame” OR “online training” OR “sports” OR “video game”) [Title/Abstract]
#3	“Anxiety” OR “depression” OR “mental health” [Title/Abstract]
#4	#1 AND #2 AND #3

### Inclusion and exclusion criteria

The PICOS principle determined eligibility criteria, including participants, intervention, comparison, outcome, and study design. Studies were included if they met the following criteria: (1) participants were diagnosed with neurological diseases; (2) the experimental group used tele-exercise intervention or game-based exercise intervention, all exercise interventions should be based on the Internet; (3) the interventions in control group include empty, usual care, general physical therapy or exercise without web-implemented methods (offline); (4) the outcomes were the common indicators reflecting depression and anxiety, such as the scores of HADS, BDI, BAI, etc. (5) only randomized controlled trials (RCTs) were selected.

Studies were excluded if they met any of the following exclusion criteria: (1) the articles were not published in English or Chinese; (2) no full-text or data was available; (3) gray literature such as conference abstracts, literature review, thesis or dissertation; (4) no web-implemented exercise intervention involved in the experimental group; (5) using web-implemented exercise interventions in the control group; (6) the intervention in the experimental group was cognitive behavior therapy or mindfulness-based intervention only.

### Study selection and data extraction

After searching the articles based on the search strategy, all studies were exported into the reference management software EndNote 20 by one researcher (HY.Z). Two researchers (R.W and M.C) independently screened the articles. After removing the duplicates and reviewing the title and abstract, the articles those are completely incompatible with the topic were deleted. Finally, two researchers (R.W and M.C) confirmed the final included studies according to the inclusion and exclusion criteria. If these two authors had an agreement and the argument was not achieved, a third arbitrator (HY.Z) would make the final determination.

Two researchers (R.W and M.C) independently extracted data from the included studies. The following information was extracted into standardized data tables: (1) study details (author, year, and country); (2) participants (sex, age, sample size, and type of patient); (3) components of interventions (types, frequency, and duration); (4) outcomes (the measurements of depression and anxiety).

### Risk of bias assessment (study quality)

Two researchers (M.C and R.W) used the Cochrane risk of bias collaboration tool to examine the study quality independently. The bias includes: (1) random sequence generation (selection bias); (2) allocation concealment (selection bias); (3) blinding of participants and personnel (performance bias); (4) blinding of outcome assessment (detection bias); (5) incomplete outcome data (attrition bias); (6) selective reporting (reporting bias); and (7) other bias. Three levels were used to evaluate the quality of included articles (i.e., low risk, high risk, and unclear risk). If the results did not match, a third researcher would make the final decision (HY.Z).

### Statistical analysis

We used Review Manager software (Review Manager 5.4) to conduct the meta-analysis. In this study, depression and anxiety were assessed using different scales. To be specific, the depression was assessed by the Hamilton depression scale (HAMD), Geriatric depression scale (GDS), Beck depression inventory (BDI), Self-rating depression scale (SDS), Depressive symptoms-9 subscale (PHQ-9), Hospital anxiety and depression (HADS) and EuroQoL (EQ-5D). The anxiety was assessed by the Hamilton anxiety scale (HAMA), beck anxiety inventory (BAI), and Self-rating anxiety scale (SAS).

Considering that the outcomes of this study were evaluated by different scales, we used standardized mean difference (SMD) to integrate the total effect size. According to the Cochrane Handbook, both the post-intervention values (Mean_post−intervention_ ± SD_post−intervention_) of the outcome and changes from baseline (Mean_changes_ ± SD_changes_) could be applied to synthesize the effect size (28). When the statistical variable was presented as the standard error (SE), the formula for calculating the standard deviation (SD) is “SE × √N.” Specifically, N is the number of subjects (29). If the statistical variable was presented as the median and quartile, the Mean and SD were calculated by the following formula:


$$\begin{array}{l} SD\approx \frac{q_{3}-q_{1}}{2\Phi^{-1}\left(\frac{0.75n-0.125}{n+0.25}\right)} \end{array}$$

(30)


$$\begin{array}{l} \bar{X}\approx \left(0.7+\frac{0.39}{n}\right)\frac{q_{1}+q_{3}}{2}+\left(0.3-\frac{0.39}{n}\right)m \end{array}$$

(31)

In two formulas above:


$$\begin{array}{l} {\text{q}}_{1}=\text{the first quartile}\\ \text{m}=\text{the median}\\ {\text{q}}_{3}=\text{the third quartile}\\ \text{n}=\text{the sample size} \end{array}$$

A compiled and published online calculator based on the formula above was provided: https://www.math.hkbu.edu.hk/~tongt/papers/median2mean.html.

If the study did not provide any of the statistical variables above, we would contact the authors via email. If we have not got a response, the study would be excluded.

In the Meta-Analysis, the effect of heterogeneity was evaluated using *I*^2^. When *I*^2^ = 0, it indicates no heterogeneity. The low, medium, and high levels of heterogeneity were represented by *I*^2^ ≤ 25%, 25%<*I*^2^ ≤ 50%, and *I*^2^ > 75%, respectively (32). The funnel plot was used to analyze publication bias. The forest plots was uesed to display the results of meta-analysis. And the sensitivity analysis was conducted to analyze the individual influence of each study on the overall result. The level of significance was set at *p* < 0.05.

## Results

### Search results

**Figure 1:**
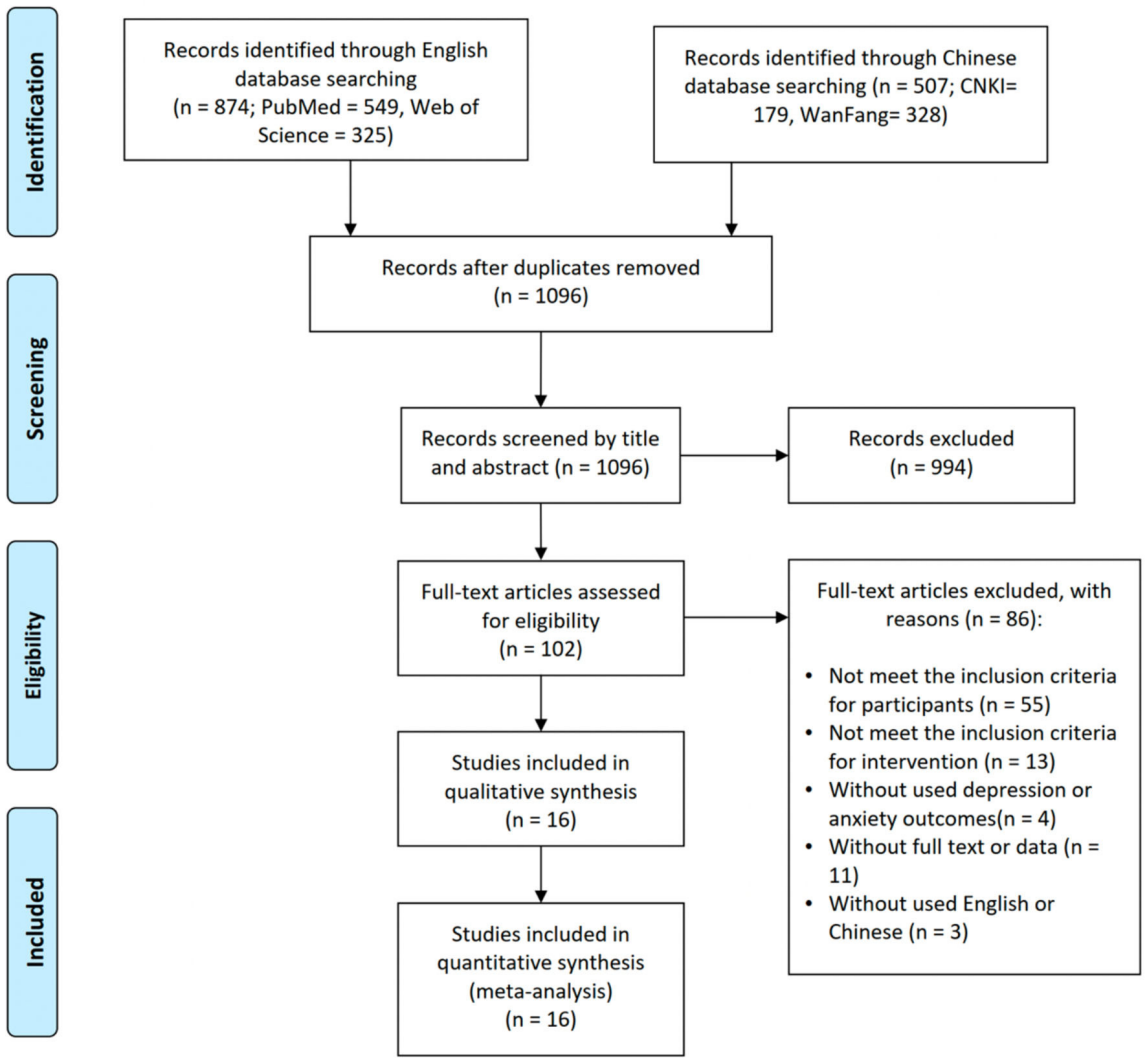
Flow diagram of the study selection process.

We identified a total of 1,381 articles, including 874 articles from the English databases and 507 articles from the Chinese databases, and 1,096 duplicates were removed. After screening the titles and abstracts, 102 articles were selected for the following full-text assessment. According to the eligibility criteria, a total of 86 articles were excluded. Among them, 55 articles did not meet the participant-related inclusion criteria, 13 articles did not meet the intervention-related inclusion criteria, four articles did not meet the outcome-related inclusion criteria, 11 articles did not provide full text or data, and three articles were not written in English or Chinese. Ultimately, a total of 16 articles were included in the meta-analysis (Figure 1).

### Study quality and bias

**Figure 2:**
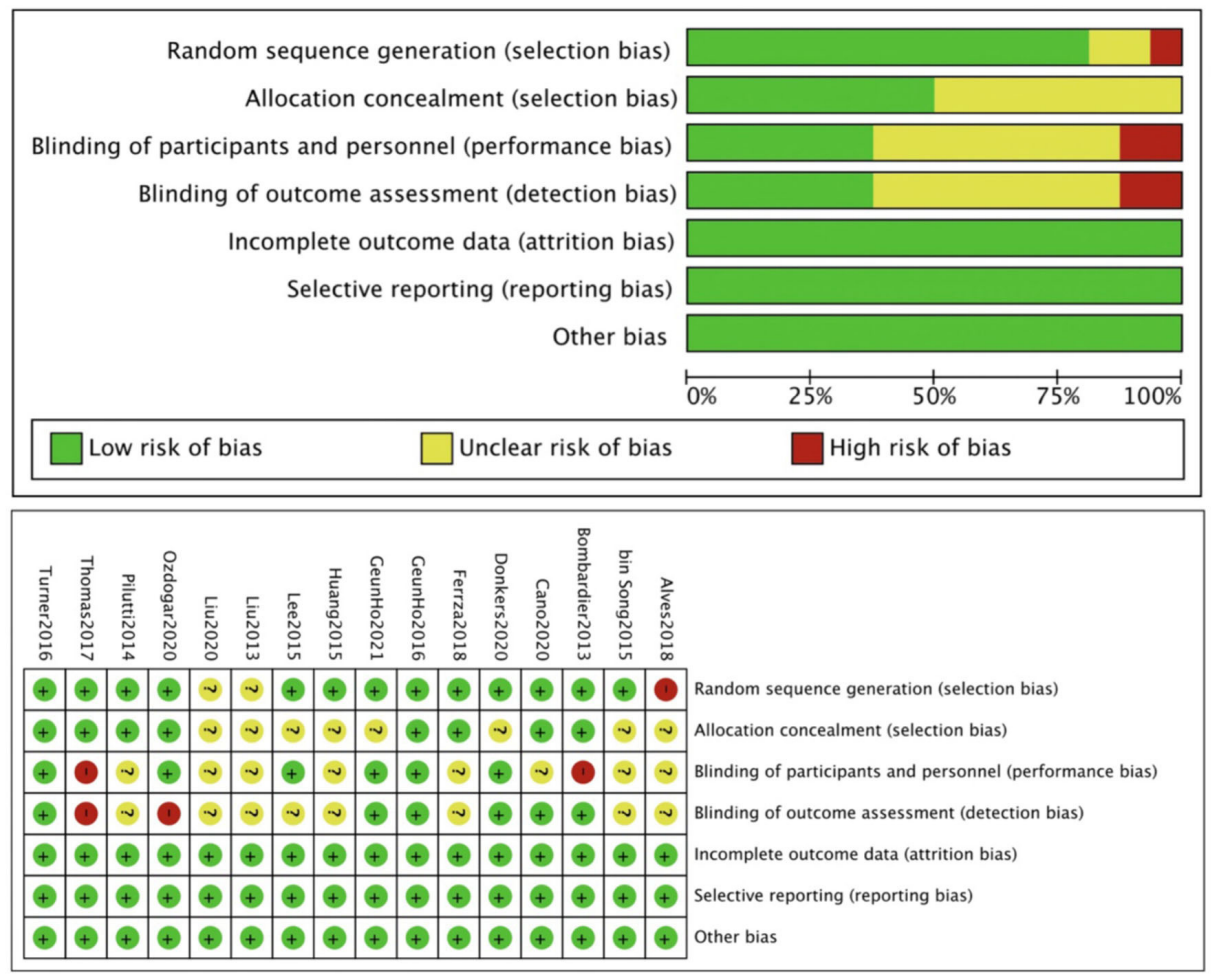
Quality assessment of included studies.

The quality assessment results of included studies were as follows (Figure 2). The majority of the studies have a low risk of bias. But the relatively high bias was performance bias and detection bias.

The sensitivity analysis was conducted using the one-by-one elimination method and the results were not affected. And the funnel plots tests did not identify any publication bias. Thus, we suggested that the pooled results were relatively stable.

### General characteristics of studies

Table 2 shows the characteristics of the selected studies, including the authors, country, participants (age, sample size, and type), interventions (type, group, frequency, and duration), and outcomes. A total of 16 studies included 963 participants from eight countries. Among these 16 studies, all patients suffered from neurological symptoms, including multiple sclerosis (MS, *n* = 6, 37.5%), cognitive impairment (*n* = 2, 12.5%), stroke (*n* = 5, 31.25%), and Parkinson's disease (PD, *n* = 3,18.75%).

**Table 2:** Characteristics of the included studies.

**References**	**Country**	**Participants**			**Different exercise interventions based on the internet**			**Outcomes (measurement)**
		**Age Mean (SD)**	**Sample size (male/female)**	**Diseases**	**Type**	**Group**	**Frequency and duration**	
Alves et al. (33)	Brazil	**EG1:** 58.89 (11.16) **EG2:** 62.67 (13.81) **CG:** 61.67 (10.74)	27 (25/2) **EG1:** 9 (9/0) **EG2:** 9 (8/1) **CG:** 9 (8/1)	Parkinson	VR exergame (Xbox and Kinect, Wii)	**EG1:** VR exergame (Will) **EG2:** VR exergame (Xbox) **CG:** without any intervention	5 weeks 45–60 min;	Anxiety: BAI
Bin Song and Cho Park (34)	South Korea	**EG:** 51.37 (4.6) **CG:** 50.1 (7.83)	40 (22/18) **EG:** 20 (10/10) **CG:** 20 (12/8)	Stroke	VR exergame (Xbox and Kinect)	**EG:** VR exergame (bowling, skiing, and golf) **CG:** bicycle training	8 weeks 30-min; 5 times/wk.	Depression: BDI
Bombardier et al. (35)	America	**EG:** 47.1 (8.9) **CG:** 49.7 (7.9)	92 (13/79) **EG:** 44 (5/39) **CG:** 48 (8/40)	Multiple sclerosis	Tele-exercise intervention	**EG:** Telephone-counseling-based physical activity **CG:** wait-list	12 weeks 30-min; telephone counseling calls in weeks 1, 2, 3, 4, 6, 8, and 10.	Depression: HAMD
Cano-Mañas et al. (36)	Spain	**EG:** 60.35 (9.84) **CG:** 65.68 (10.68)	48 (23/25) **EG:** 23 (12/11) **CG:** 25 (11/14)	Stroke	VR exergame (Xbox and Kinect)	**EG:** VR exergame (20-min) (tennis, baseball, etc.) + conventional rehabilitation (35 min physical + 35 min occupational therapy) **CG:** conventional rehabilitation (single leg support, standing with assistance and autonomy, etc.)	8 weeks **EG:** 3 times/wk. **CG:** conventional rehabilitation (45 min physical + 45 min occupational therapy)	Depression and anxiety: EQ-5D
Geun-Ho (37)	South Korea	**EG:** 63.8 (10.2) **CG:** 65.5 (8.1)	30 (18/12) **EG:** 15 (10/5) **CG:** 15 (8/7)	Cognitive impairment (early dementia)	VR exergame (Wii-fit and Wii)	**EG:** VR exercise program [30-min Will Fit balance game + 10-min Wii sports game (golf or bowling)] + cognitive rehabilitation **CG:** Cognitive rehabilitation program	12 weeks 36 sessions **EG:** 40-min; 3 times/wk. **CG:** 20-min/sessions	Depression: GDS-K
Geun-Ho (12)	South Korea	**EG:** 71.45 (6.33) **CG:** 72.12 (6.48)	40 (30/10) **EG:** 20 (13/7) **CG:** 20 (12/8)	Cognitive impairment (early dementia)	VR exergame (Wii)	**EG:** VR exergame (fencing, bowling, table tennis, and frisbee) **CG:** Usual care	12 weeks 15 min; 3 times/wk.	Depression: GDS-K
Huang and Xiaotong (38)	China	**EG:** 65.8 (8.01) **CG:** 66.4 (8.16)	60 (35/25) **EG:** 30 (17/13) **CG:** 30 (18/12)	Stroke	Tele-exercise intervention	**EG:** Video conference (live face-to-face): Brunnstrom training (walking, sit and stand balance, the upper and lower limbs lift and fall) + Medical and psychological guidance **CG:** Usual care	6 months 45-min; In the 1st month, 1 time/week; in the 2–6-month, 1 time/2 week.	Anxiety: HAMA; Depression: HAMD.
Lee et al. (27)	South Korea	**EG:** 68.4 (2.9) **CG:** 70.1 (3.3)	20 (10/10) **EG:** 10 (5/5) **CG:** 10 (5/5)	Parkinson	VR exergame (Wii)	**EG:** VR dance exergame (K-pop dance festival) + neurodevelopment treatment (NDT) + functional electrical stimulation (FES) **CG:** NDT + FES	6 weeks 5 times/wk. **EG:** 30-min exergame + 30-min NDT + 15-min FES **CG:** 30-min NDT + 15-min FES	Depression: BDI
Liu (39)	China	**EG:** 52.93 (6.2) **CG:** 51.67 (7.18)	60 (46/14) **EG:** 30 (24/6) **CG:** 30 (22/8)	Stroke	VR exergame (Kinect)	**EG:** VR exergame (skiing) + physiotherapy **CG:** Regular exercise + physiotherapy	4 weeks 30-min; 3 times 4 week	Depression: SDS
Liu (40)	China	**EG:** 61.36 (7.81) **CG:** 63.15 (8.46)	200 (117/83) **EG:** 100 (61/39) **CG:** 100 (56/44)	Stroke	Tele-exercise intervention	**EG:** Functional training (live face- to-face video conferencing, App) + Medical and psychological guidance **CG:** Usual care	3 months 60-min; 5 times/wk.	Anxiety: SAS Depression: SDS
Ozdogar et al. (41)	Turkey	**EG:** 39.2 (8.6) **CG1:** 43.6 (10.5) **CG2:** 37.9 (12.4)	60 (16/44) **EG:** 21 (5/16) **CG1:** 19 (6/12) **CG2:** 20 (5/15)	Multiple sclerosis	VR exergame (Xbox and Kinect)	**EG:** Kinect sports rivals' game (bowling, Jet Ski racing, rock climbing, football, tennis, and target shooting) **CG1:** Conventional rehabilitation in the center (balance, arm, and core stability exercise) **CG2:** wait-list	8 weeks 45-min, 1 time/wk.	Depression: BDI
Pilutti et al. (42)	America	**EG:** 48.4 (9.1) **CG:** 49.5 (9.2)	82 (20/62) **EG:** 41 (11/30) **CG:** 41 (9/32)	Multiple sclerosis	Tele-exercise intervention	**EG:** Telehealth monitoring, web-implemented video scheduled **CG:** wait-list	6 months Total 15 web-implemented video coaching sessions	Depression and anxiety: HADS
Thomas et al. (43)	Britain	**EG:** 50.9 (8.08) **CG:** 47.6 (9.26)	30 (3/27) **EG:** 15 (1/14) **CG:** 15 (2/13)	Multiple sclerosis	VR exergame (Wii)	**EG:** VR exergame **CG:** wait-list	6 months	Depression and anxiety: HADS
Turner et al. (26)	America	**EG:** 52.7 (11.6) **CG:** 53.6 (13.1)	64 (41/23) **EG:** 31 (22/9) **CG:** 33 (19/14)	Multiple sclerosis	Tele-exercise intervention	**EG:** Telephone counseling (TC + DVD) + home telehealth monitoring **CG:** Self-directed education (DVD)	6 months **EG:** TC in 6 times/wk.; suggestion: moderate-intensity activity; 45-min; 1–2 times/wk.	Depression: PHQ-9
Ferraz et al. (44)	Brazil	**G1:** 68.57 (9.92) **G2:** 64.66 (13.1) **G3 (EG):** 67.76 (14.45)	62 (37/25) **G1:** 22 (16/6) **G2:** 20 (11/9) **G3 (EG):** 20 (10/10)	Parkinson	VR exergame (Xbox and Kinect)	**G1:** Functional group **G2:** Bike training group **G3 (EG):** Exergaming group	8 weeks 50-min; 3 times/wk.	Depression: GDS-15
Donkers et al. (45)	Canada	**EG:** 54.6 (11.9) **CG:** 53.8 (12.2)	48 (17/31) **EG:** 32 (12/20) **CG:** 16 (5/11)	Multiple sclerosis	Tele-exercise intervention	**EG:** Website contains exercise (video, text, and audio descriptions) **CG:** Usual care	6 months 2 times/wk.;	Depression and anxiety: HADS

EG, experimental group; CG, control group; VR, virtual reality; HAMA, Hamilton anxiety scale; HAMD, Hamilton depression scale; PHQ-9, Depressive symptoms-9 subscale; SAS, Self-rating anxiety scale; SDS, Self-rating depression scale; GDS-K, Geriatric depression scale-Korean; BDI, Beck depression inventory; wait-list, participant were told that baseline testes need to be repeated after a certain amount of time, and will have opportunity to receive intervention; HADS, Hospital anxiety and depression. EQ-5D, Self-administered questionnaire, evaluated the QOL in five dimensions (including depression and anxiety); BAI, Beck anxiety inventory; GDS-15, 15-item geriatric depression scale.

Web-implemented exercise interventions were separated into two main categories (i.e., tele-exercise intervention and VR exergame), respectively (Wii, Xbox and Kinect). There are six studies (37.5%) involving tele-exercise intervention and 10 studies (62.5%) involving VR exergame (Wii, Xbox, and Kinect). In addition, according to the concrete contents of interventions in the experimental group, 10 studies (62.5%) conducted single-component interventions (only web-implemented exercise) and six studies (37.5%) conducted multi-component interventions (e.g., the exercise intervention combined with psychological, physical therapy, and some other interventions). In terms of outcomes, 15 studies (93.8%) involved depression and seven studies (43.8%) involved anxiety.

### Effect of web-implemented exercise interventions on depression and anxiety

**Figure 3:**
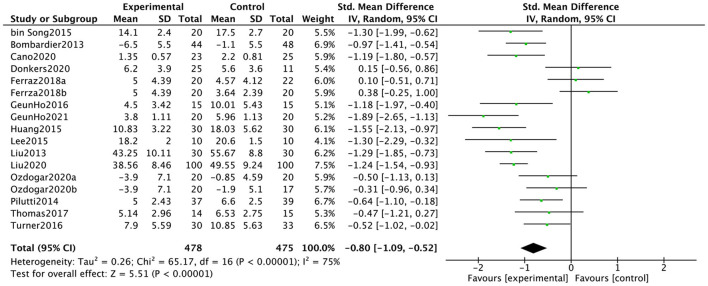
Effect of web-implemented exercise interventions on depression.

Seventeen data points from 15 studies reported the influence of web-implemented exercise interventions on depression in patients with neurological symptoms. Figure 3 showed that the web-implemented exercise intervention has a significant effect on depression in patients with neurological symptoms. Based on a random-effect model, the pooled effect size was SMD = −0.80 (95% CI, −1.09 to −0.52). The heterogeneity was high and significant (*I*^2^ = 75%, *p* < 0.00001).

**Figure 4:**
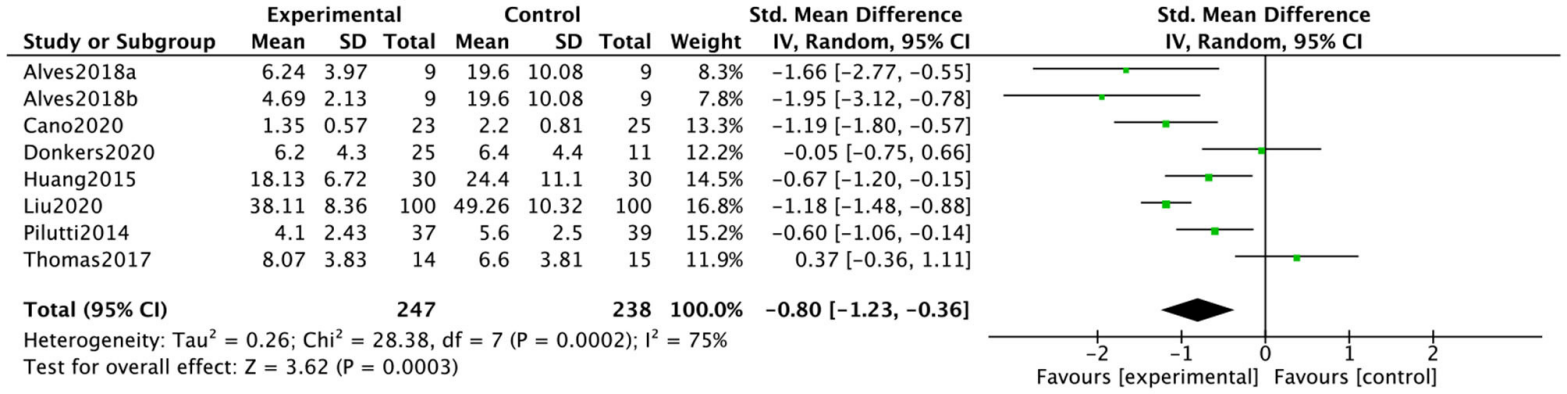
Effect of web-implemented exercise interventions on anxiety.

Eight data points from seven studies reported the influence of web-implemented exercise interventions on anxiety in patients with neurological symptoms. Figure 4 showed that the web-implemented exercise intervention has a significant effect on anxiety in patients with neurological symptoms. Based on a random-effect model, the pooled effect size was SMD = −0.80 (95% CI, −1.23 to −0.36). The heterogeneity was high and significant (*I*^2^ = 75%, *p* = 0.0003).

### Subgroup analysis

We performed subgroup analysis based on the type of interventions, type of patients, duration of interventions, and intervention components. Table 3 showed the subgroup analysis of the effect of web-implemented exercise interventions on depression. For intervention types, based on a random-effect model, both tele-exercise (SMD = −0.83, 95% CI, −1.23 to −0.43, *I*^2^ = 77%, *P* < 0.0001) and VR exergame (SMD = −0.79, 95% CI, −1.21 to −0.37, *I*^2^ = 76%, *P* = 0.0002) had a significant relieving effect on depression. According to the effect size, the tele-exercise interventions appeared to have a better-alleviating effect on depression than VR exergame interventions. For contents of tele-exercise intervention, both face-to-face intervention (SMD = −1.30, 95% CI, −1.57 to −1.03, *I*^2^ = 0%, *P* < 0.00001) and offline delayed interventions (SMD = −0.56, 95% CI, −0.96 to −0.16, *I*^2^ = 59%, *P* = 0.006) had a significant effect on reducing depression. For the components of interventions in the experimental group, both single-component (only web-implemented exercise) intervention (SMD = −0.54, 95% CI, −0.89 to −0.19, *I*^2^ = 73%, *P* = 0.003) and multi-component (the web-implemented exercise intervention combined with other interventions) interventions (SMD = −1.28, 95% CI, −1.49 to −1.07, *I*^2^ = 0%, *P* < 0.00001) had a significant effect on reducing depression. According to the effect size, the multi-component interventions had a better effect than the single-component intervention. Based on the difference in interventions in the control group, we divided the control group into the empty control group (e.g., usual care and wait-list) and the offline-exercise control group. Compared with the empty control group (SMD = −0.89, 95% CI, −1.22 to −0.56, *I*^2^ = 71%, *P* < 0.00001) and offline-exercise control group (SMD = −0.68, 95% CI, −1.22 to −0.13, *I*^2^ = 80%, *P* = 0.01) separately, the web-implemented exercise interventions in the experimental group had a significant relieving effect on decreasing depression. However, when compared with the empty control group, the web-implemented exercise interventions in the experimental group had a better effect. When it comes to the intervention duration, the results of the sub-group analysis showed that the effect of the short-term (i.e., <6 weeks) web-implemented exercise intervention (SMD = −1.30, 95% CI, −1.78 to −0.81, *I*^2^ = 0%, *P* < 0.00001) was better than that of the medium-term (i.e., 6–12 weeks) web-implemented exercise intervention (SMD = −0.80, 95% CI, −1.21 to −0.40, *I*^2^ = 80%, *P* < 0.0001) and the long-term (i.e., more than 12 weeks) web-implemented exercise intervention (SMD = −0.63, 95% CI, −1.13 to −0.13, *I*^2^ = 73%, *P* = 0.01). For patients with different neurological diseases, the results showed that web-implemented exercise interventions had a significant effect on depression among patients with stroke (SMD = −1.29, 95% CI, −1.50 to −1.07, *I*^2^ = 0%, *P* < 0.00001), MS (SMD = −0.54, 95% CI, −0.79 to −0.29, *I*^2^ = 27%, *P* < 0.0001) and Cognitive impairment (SMD = −1.54, 95% CI, −2.24 to −0.85, *I*^2^ = 38%, *P* < 0.0001). No significant effect was observed in patients with PD (SMD = −0.19, 95% CI, −1.03 to 0.65, *I*^2^ = 76%, *P* = 0.66).

**Table 3:** Subgroup analysis of the effect of web-implemented exercise interventions on depression.

**Subgroups**	***N***	***n***	***SMD***	**95% *CI***	***Z***	***I^2^***
**Type of intervention**	17	953	−0.80	−1.09 to −0.52	5.51^**^	75%^**^
Tele-exercise	6	527	−0.83	−1.23 to −0.43	4.10^**^	77%^**^
VR exergame	11	426	−0.79	−1.21 to −0.37	3.70^**^	76%^**^
**Contents of tele-exercise**	6	527	−0.83	−1.23 to −0.43	4.10^**^	77%^**^
Face-to face	2	260	−1.30	−1.57 to −1.03	9.51^**^	0%
Offline delayed	4	267	−0.56	−0.96 to −0.16	2.74^**^	59%
**Component**	17	953	−0.80	−1.09 to −0.52	5.51^**^	75%^**^
Single-component	11	535	−0.54	−0.89 to −0.19	3.01^**^	73%^**^
Multi-component	6	418	−1.28	−1.49 to −1.07	11.84^**^	0%
**Control group**	17	953	−0.80	−1.09 to −0.52	5.51^**^	75%^**^
Therapy or exercise	7	656	−0.68	−1.22 to −0.13	2.43^*^	80%^**^
Empty	10	297	−0.89	−1.22 to −0.56	5.31^**^	71%^**^
**Type of patients**	17	953	−0.80	−1.09 to −0.52	5.51^**^	75%^**^
PD	3	102	−0.19	−1.03 to 0.65	0.44	76%^*^
Stroke	5	408	−1.29	−1.50 to −1.07	11.77^**^	0%
MS	6	373	−0.54	−0.79 to −0.29	4.22^**^	27%
CI	2	70	−1.54	−2.24 to −0.85	4.36^**^	38%
**Duration (wks.)**	17	953	−0.80	−1.09 to −0.52	5.51^**^	75%^**^
<6	2	80	−1.30	−1.78 to −0.81	5.22^**^	0%
6–12	10	609	−0.80	−1.21 to −0.40	3.91^**^	80%^**^
>12	5	264	−0.63	−1.13 to −0.13	2.47^*^	73%^**^

N, the number of included studies; n, sample size; SMD, Standardized Mean Difference; CI, confidence interval; MS, multiple sclerosis; CI, Cognitive impairment; PD, Parkinson's disease.

* *p* < 0.05.

** *p* < 0.01.

Table 4 showed the subgroup analysis of the effect of web-implemented exercise interventions on anxiety. For intervention types, based on a random-effect model, both tele-exercise (SMD = −0.69, 95% CI, −1.15 to −0.23, *I*^2^ = 73%, *P* = 0.003) and VR exergame (SMD = −1.05, 95% CI, −2.08 to −0.01, *I*^2^ = 83%, *P* = 0.05) had a significant relieving effect on anxiety. According to the effect size, the VR exergame interventions appeared to have a better-alleviating effect on anxiety than tele-exercise interventions. For contents of tele-exercise intervention, both face-to-face intervention (SMD = −0.98, 95% CI, −1.46 to −0.51, *I*^2^ = 62%, *P* < 0.0001) and offline delayed interventions (SMD = −0.39, 95% CI, −0.92 to 0.14, *I*^2^ = 40%, *P* = 0.15) had a significant effect on reducing anxiety. For the components of interventions in the experimental group, both single-component (only web-implemented exercise) intervention (SMD = −0.62, 95% CI, −1.29 to 0.06, *I*^2^ = 78%, *P* = 0.08) and multi-component (the web-implemented exercise intervention combined with other interventions) interventions (SMD = −1.05, 95% CI, −1.36 to −0.73, *I*^2^ = 31%, *P* < 0.00001) had a significant effect on reducing anxiety. According to the effect size, the multi-component interventions had a better effect than the single-component intervention. In terms of the intervention duration, the results of the sub-group analysis showed that the effect of the short-term (i.e., <6 weeks) web-implemented exercise intervention (SMD = −1.80, 95% CI, −2.60 to −0.99, *I*^2^ = 0%, *P* < 0.00001) was better than that of the medium-term (i.e., 6–12 weeks) web-implemented exercise intervention (SMD = −1.18, 95%CI, −1.45 to −0.91, *I*^2^ = 0%, *P* < 0.00001) and the long-term (i.e., more than 12 weeks) web-implemented exercise intervention (SMD = −0.30, 95% CI, −0.76 to 0.15, *I*^2^ = 57%, *P* = 0.19). For patients with different neurological diseases, the results showed that web-implemented exercise interventions had a significant effect on anxiety among patients with PD (SMD = −1.80, 95% CI, −2.60 to −0.99, *I*^2^ = 0%, *P* < 0.0001) and strokes (SMD = −1.05, 95% CI, −1.36 to −0.73, *I*^2^ = 31%, *P* < 0.00001). No significant effect was observed in patients with MS (SMD = −0.15, 95% CI, −0.74 to 0.44, *I*^2^ = 62%, *P* = 0.62). Considering that only one study conducted the offline-exercise intervention in the control group, other studies were all blank controls, so we did not perform subgroup analyses based on the difference of interventions in the control group.

**Table 4:** Subgroup analysis on anxiety.

**Subgroups**	***N***	***n***	***SMD***	**95% *CI***	***Z***	***I^2^***
**Type of intervention**	8	485	−0.80	−1.23 to −0.36	3.62^**^	75%^**^
Tele-exercise	4	372	−0.69	−1.15 to −0.23	2.97^**^	73%
VR exergame	4	113	−1.05	−2.08 to −0.01	1.98	83%^**^
**Contents of tele-exercise**	4	372	−0.69	−1.15 to −0.24	2.99^**^	73%^*^
Face-to face	2	260	−0.98	−1.46 to −0.51	4.03^**^	62%
Offline delayed	2	112	−0.39	−0.92 to 0.14	1.45^**^	40%
**Component**	8	485	−0.80	−1.23 to −0.36	3.62^**^	75%^**^
Single–component	5	177	−0.62	−1.29 to 0.06	1.78	74%^**^
Multi–component	3	308	−1.05	−1.36 to −0.73	6.54^**^	31%
**Type of patients**	8	485	−0.80	−1.23 to −0.36	3.62^**^	75%^**^
PD	2	36	−1.80	−2.60 to −0.99	4.37^**^	0%
Stroke	3	308	−1.05	−1.36 to −0.73	6.54^**^	31%
MS	3	141	−0.15	−0.74 to 0.44	0.49	74%
**Duration (wks.)**	8	485	−0.80	−1.23 to −0.36	3.62^**^	75%^**^
≤6	2	36	−1.80	−2.60 to −0.99	4.37^**^	0%
6–12	2	248	−1.18	−1.45 to −0.91	8.57^**^	0%^**^
>12	4	201	−0.30	−0.76 to 0.15	1.32	57%

N, the number of included studies; n, sample size; SMD, Standardized Mean Difference; CI, confidence interval; MS, multiple sclerosis; PD, Parkinson's disease.

* *p* < 0.05.

** *p* < 0.01.

## Discussion

This systematic review and meta-analysis has evaluated the effects of web-implemented exercise interventions on depression and anxiety in patients with neurological disorders by integrating 16 scientific studies involving a total of 963 participants. The review has shown that web-implemented exercise intervention significantly improves depression and anxiety in patients with neurological disorders. In addition, the effectiveness of the web-implemented exercise intervention is influenced by several factors, including web-implemented exercise intervention type, component, intervention duration, and patients with different neurological diseases. In terms of the type of web-implemented exercise interventions, the effect of tele-exercise is better than that of VR exergame on depression. While in relieving anxiety, the effect of VR exergame is better than that of tele-exercise. For the components of web-implemented exercise intervention, the effect of the multi-component intervention is better than that of the single-component on depression and anxiety. Additionally, based on the duration-related subgroup analysis, the effects of the web-implemented exercise intervention on both depression and anxiety were worse when the duration of interventions extends.

Our result has demonstrated that web-implemented exercise interventions have a significant effect on the reduction of depression and anxiety in patients with neurological disorders. Exercise, as a non-medicine and non-invasive intervention, has been proven to reduce depression and anxiety in several RCTs (46, 47). It has been proved that a loss of dopamine and norepinephrine innervation in the limbic system can lead to increased depression and anxiety disorder in patients with certain neurological diseases (48). Exercise has been demonstrated to alter the monoamine levels (e.g., serotonin, dopamine, and norepinephrine) and stress hormone cortisol to maintain mood (49, 50). On the other hand, there is some evidence showing that exercise can promote neuroprotection through molecular adaptations (e.g., the activation of the PGC-1α/FNDC5/Irisin pathway), leading to maintaining good mental health (51, 52). In consequence, neurological disorders patients may obtain huge benefits from exercise interventions for depression and anxiety. However, it is well-known that pain, fatigue, and the lack of energy induced by the neurological disease may make patients with neurological disorders tend to perform few interests in daily physical activities, even exercise. Hence, traditional exercise interventions may be inapplicable to patients to alleviate depression and anxiety. The exercise interventions with interactive communication and exergame based on virtual reality (VR) technology, video, and some other web-implemented media may help patients be more interested in exercise by allowing participants to immerse themselves in the “digital and interactive world” (53–56). In addition, the web-implemented methods can make patients exercise at home with supervision and develop targeted and personalized training. The web-implemented exercise intervention, as a complementary and alternative exercise intervention, can be used to replace or replenish traditional exercise and rehabilitation medicine, which are relatively inexpensive, time-saving, and spatial flexible. For patients with neurological disorders who have neurological dysfunction and motor system problems, web-implemented exercise interventions are more convenient and efficient than traditional exercise (57, 58). Therefore, web-implemented exercise intervention has a remarkable potential to decrease depression and anxiety in patients with neurological disorders.

The subgroup analysis in our meta-analysis has also indicated that different types of web-implemented exercise interventions have different anesis degrees on depression and anxiety in patients with neurological disorders. The tele-exercise guidance seems to have a better effect on depression than VR exergames. While the VR exergame seems to have a better effect on anxiety than tele-exercise intervention. Although the difference between VR exergame and tele-exercise in our meta-analysis was not significant (depression: *P* = 0.89; anxiety: *P* = 0.54) and the fact that depression and anxiety may occur together make it difficult to distinguish the effect of web-implemented exercise interventions on depression and anxiety separately (59, 60). The trend of difference between the effect of these two web-implemented exercise interventions on depression and anxiety can be discriminated by the effect size (i.e., SMD) of depression (tele-exercise: −0.69 vs. VR exergame: −1.05) and anxiety (tele-exercise: −0.83 vs. VR exergame: −0.79). According to the clinical characteristics of depression and anxiety, the difference may be explained. The symptoms of depressive disorders are mainly reflected in the sense of hopelessness, sadness, repression, and fear of embarrassment (59). Tele-exercise, an online exercise intervention based on the actual communication between individuals, can help patients alleviate depression-related complications through the face-to-face phone- and web-meetings. A targeted and personalized training plan will be conveyed to patients through a real conversation, which makes patients and exercise professionals communicate timely. The professional can give positive information and promote patients with depressive symptoms to produce an optimistic mood (61, 62). Hence, specific online communication may be an advantage in tele-exercise intervention, while VR exergame interventions cannot provide effective interpersonal communication. On the contrary, the characteristics of anxiety disorders were being tense, extreme panic, phobia, and misery (63). Compared to tele-exercise interventions, VR exergames can stabilize and ease patients' anxious emotions by immersing them in exercise games and increasing their autonomy and interest (64). The unique online competition and recreation features of VR exergame and immersive experience may alleviate restlessness and fretfulness caused by anxiety (37). Except that, various sports exercise modes (e.g., golf, bowling, skiing, fencing, rock climbing, shooting, soccer, dancing, tennis, and Frisbee) of VR exergames can bring more choices for patients with different exercise interests to relieve anxiety and maintain a positive mood (39, 65).

We further compared tele-exercise and VR exergame two intervention methods. First, according to the contents of tele-exercise intervention, we found that both web- implemented face-to-face intervention and offline delayed intervention had positive effect on anxiety and depression. Second, we summarized the frequency and intensity of all the studies and found that almost all studies recommend using moderate to high intensity exercise, about 20–60 min/time, 2–5 times/week, and an average of about 100–180 min/week. One meta-analysis of stroke found that high-intensity exercise had a significant effect on depression, while low intensity had no effect (66). Another study also suggests that moderate-intensity exercise may be an optimal intensity when using exercise as a treatment for mental health (67). VR exergame can adjust the intensity of the exercise by adjusting the difficulty and mode of tasks and have high-level repeatability (27, 36). Tele-exercise interventions used personalized to tailor the intensity of exercise to the patient's situation. Even if some patients were not achieving the target intensity, it is recommended that the intensity of exercise can be gradually increased from low to high, and that they will be encouraged to actively participate in exercise and increase their daily physical activity (26, 35).

For the components of web-implemented exercise interventions, our results have demonstrated that the multi-component web-implemented exercise intervention (e.g., the random combination of psychological therapy, physical therapy, neurological therapy, functional electrical therapy, and cognitive rehabilitation therapy) has a better effect than the single-component web-implemented exercise intervention (e.g., tele-exercise or VR exergame only) on reducing both depression and anxiety symptoms in neurological patients. We have found that three studies involving single-exercise component intervention show no significant relieving effect on depression and anxiety symptoms in the experimental group, or even a lower effect in the experimental group than that in the control group (43–45). In contrast, all studies using multi-component interventions appear that the effect of alleviating depression and anxiety is better in the experimental group than that in the control group. For patients with neurological disorders, the primary purpose of web-implemented exercise intervention is to restore physical function and improve QOL, which can indirectly relieve depression and anxiety by restoring physical function and reducing disease symptoms. However, other medical and psychological interventions like physical therapy, neurological therapy, functional electrical therapy, psychological therapy, and cognitive rehabilitation therapy are also beneficial for the decrease of depression and anxiety as well (36, 38, 41, 44). Thus, the multi-component intervention may have double and multiple effects to alleviate depression and anxiety.

Based on the duration-related subgroup analysis, the result has indicated that the effect of the web-implemented exercise intervention on reducing depression and anxiety became less effective with the extension of intervention duration. This result is contrary to the results of a previous relevant meta-analysis. Huang et al. (68) have found that long-term interventions were more effective in relieving depression. These opposite results may be due to the difference of web-implemented exercise intervention types and sample types. Huang et al. have integrated the effect of isolated VR exergame on depression of a mixed sample including healthy adults and various patients (e.g., MS, stroke, PD, and hemodialysis). Otherwise, our study has merged the effect of the web-implemented exercise interventions consisting of VR exergame and tele-exercise on depression and anxiety in patients with neurological disorders. We have speculated that the population with neurological disorders has better compliance and adherence to the interventions than the healthy population. Thus, compared to a mixed sample, the presence of the effect of web-implemented exercise intervention in patients with neurological diseases does not need to take a too long time.

The meta-analysis still has some limitations. First, most studies used depression and anxiety as the secondary outcomes, which might weaken the causal relationship between the web-implemented exercise intervention and depression and anxiety. Thus, further research is needed to directly prove the relationship between web-implemented exercise intervention and mental health outcomes (e.g., depression and anxiety). Second, this meta-analysis has included a variety of characteristics of web-implemented exercise interventions (e.g., intervention type and intervention dosages). The included studies involved different participant races and study designs, which may lead to moderate to high heterogeneity and lower quality. Finally, the limited number of included studies and the language restrictions may also cause bias on the integrated effect.

## Conclusion

This meta-analysis indicates that web-implemented exercise intervention can significantly relieve depression and anxiety symptoms in patients with neurological disorders. The current results suggest that short-term (≤6 weeks) and multi-component web-implemented exercise interventions have the better effect. In addition, tele-exercise has a better-relieving effect on depression, while VR exergame has a better-relieving effect on anxiety. It should be noted that the web-implemented exercise intervention is only a form of clinical therapy support. Except that, due to limitations and scarcity of studies involving the impact of web-implemented exercise interventions on mental health in neurological patients, more relevant studies are needed in the future. It is suggested to increase long-term follow-up and study biological mechanisms to further evaluate the effect of the web-implemented exercise intervention on mental health in neurological patients.

## Data availability statement

The original contributions presented in the study are included in the article/supplementary material, further inquiries can be directed to the corresponding authors.

## Author contributions

SZ and XH: conceptualization and supervision. HZ and RW: methodology. HZ: formal analysis and writing—original draft preparation. HZ, RW, and ZK: investigation. ZK and JY: resources. HZ, RW, and XH: writing—review and editing. HZ and SZ: visualization. All authors listed have made a substantial, direct, and intellectual contribution to the work and approved it for publication.
